# Supplementary material for: Reconfiguring crystal and electronic structures of MoS2 by substitutional doping
Source: Nat Commun. 2018 Jan 15;9:199. doi: 10.1038/s41467-017-02631-9 (PMC5768716; doi:10.1038/s41467-017-02631-9)
Supplement: Supplementary file 1 — Supplementary Information [file 41467_2017_2631_MOESM1_ESM.pdf]

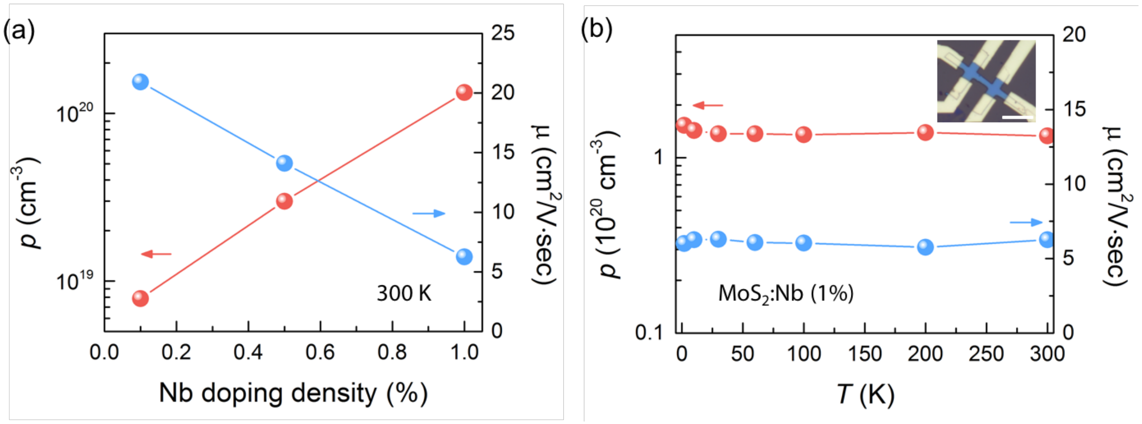

Supplementary Figure 1 │ (a) Room-temperature hole concentration and mobility as a function of Nb doping density. Hall-effect data for 0.5% Nb doping were taken from Ref #6. **(b)** Temperature dependent carrier concentration and mobility for 1% Nb-doped MoS_2. Inset scale bar, 10 μm.

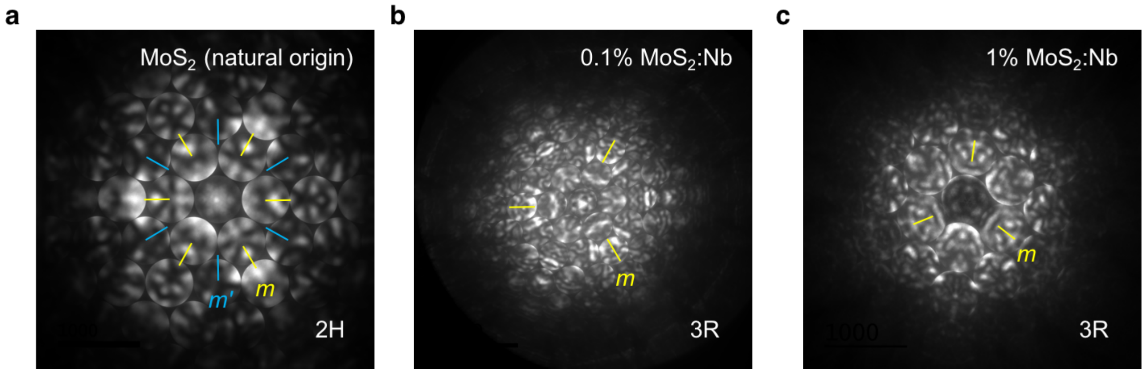

Supplementary Figure 2 │ Additional CBED patterns acquired from commercially available MoS_2 (natural origin) (**a**), and 0.1% and 1% Nb-doped MoS_2 crystals (**b,c**).

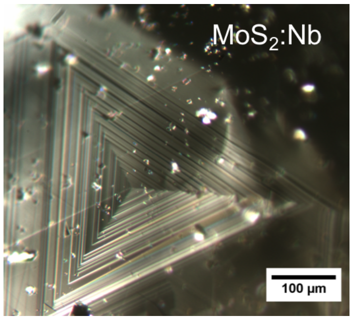

Supplementary Figure 3│ Optical microscopy image of a screw dislocation spiral. Optical micrograph taken from the surface of Nb-doped MoS_2 (0.5%) crystal displaying a clear triangular feature as previously observed in undoped 3R-MoS_2[1]. We note, however, that the MoS_2:Nb simultaneously shows the mixed character of triangular and hexagonal spirals, possibly implying that 2H and 3R may co-exist in MoS_2:Nb.

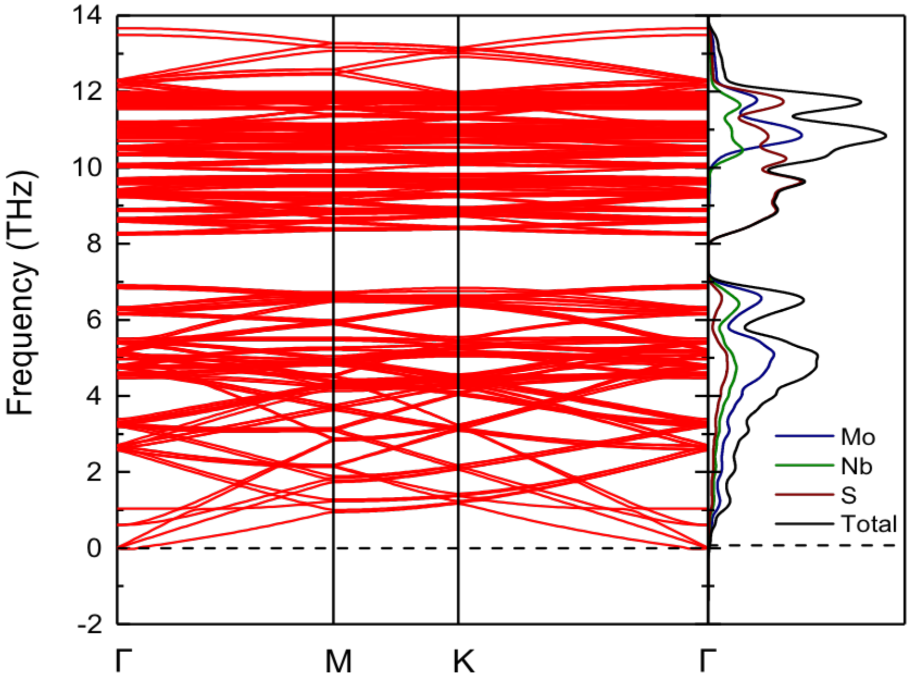

Supplementary Figure 4 │ Phonon dispersion and corresponding phonon density of states (DOS) of the Nb-doped 3R-MoS_2 supercell. The phonon calculations of the relaxed Nb-doped MoS_2 4 × 4 supercell were carried out using the PHONOPY code[2], using the finite atomic displacements method with an amplitude of 0.01 Å to obtain the atomic forces within the supercell. This is followed by the dynamical matrix approach to acquire the phonon frequencies.

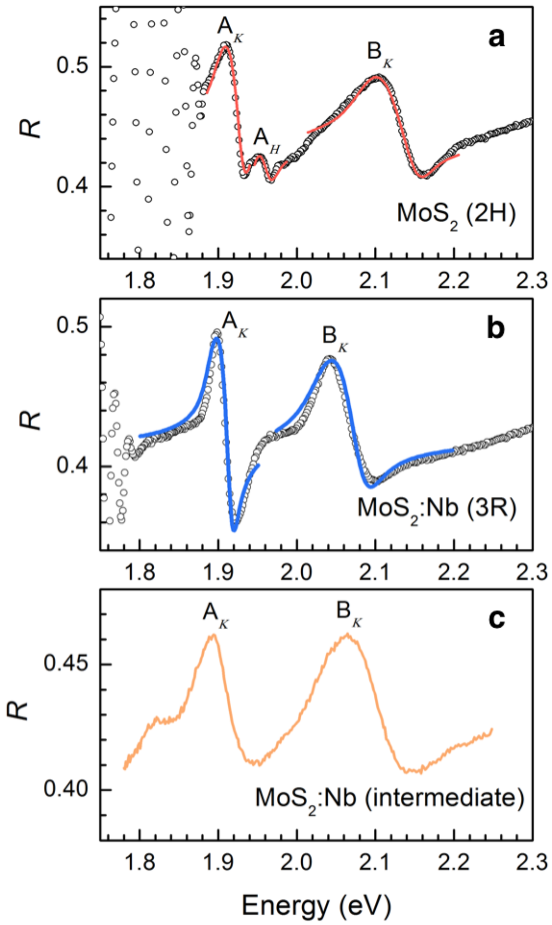

Supplementary Figure 5│ Micro-reflectance spectra measured from undoped MoS_2 (**a**) and Nb-doped MoS_2 (**b**) crystals. Distinctive intermediate phase was also observed from some local areas of Nb-doped MoS_2 (**c**). All spectra were taken at 4.5 K.

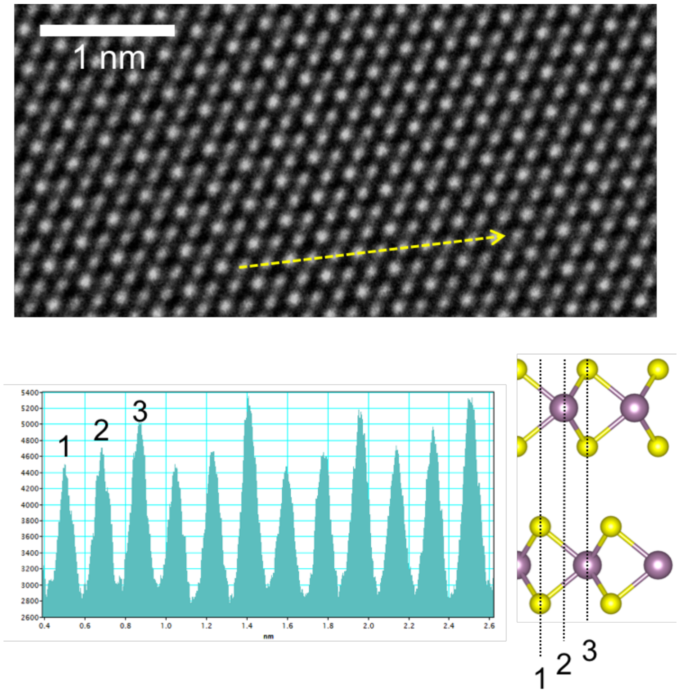

Supplementary Figure 6│3R-type bilayer Nb-doped MoS_2. (Top panel) High-resolution transmission electron microscopy image of bilayer Nb-doped MoS_2, clipped from Fig. 2a. (Bottom panels) Corresponding line intensity profile and illustration of atomic model of 3R bilayer. A hexagonal pattern with the measured 1.82 Å lattice spacing is consistent with earlier reports[3,4].

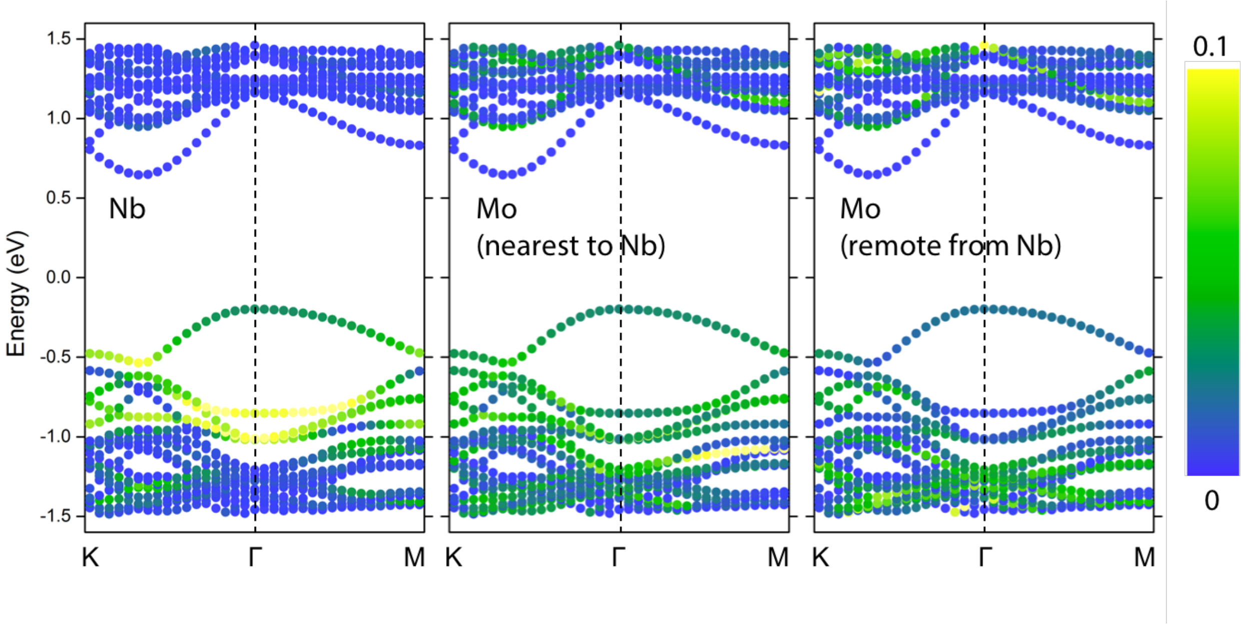

Supplementary Figure 7 | Band structures of bilayer 3R Nb-doped MoS_2 projected onto the Nb and Mo atoms. For Mo, the projections are made on two atoms with different distances from Nb dopant for comparison. A 4 × 4 supercell is used, and it is found that $E_F = -0.35$ eV. The color scale indicated the magnitude of the projection.

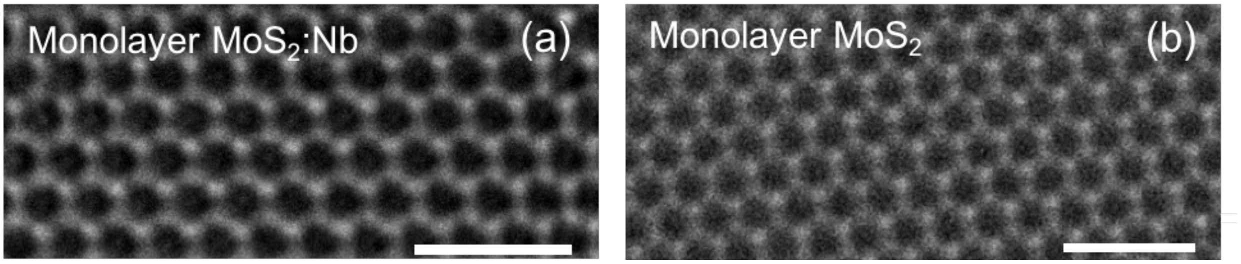

Supplementary Figure 8 | Spherical aberration-corrected high-resolution transmission electron microscopy (HRTEM) images with sub-Å resolution acquired from Nb-doped (**a**) and Nb-free (**b**) monolayers, respectively. Both scale bars correspond to 1 nm.

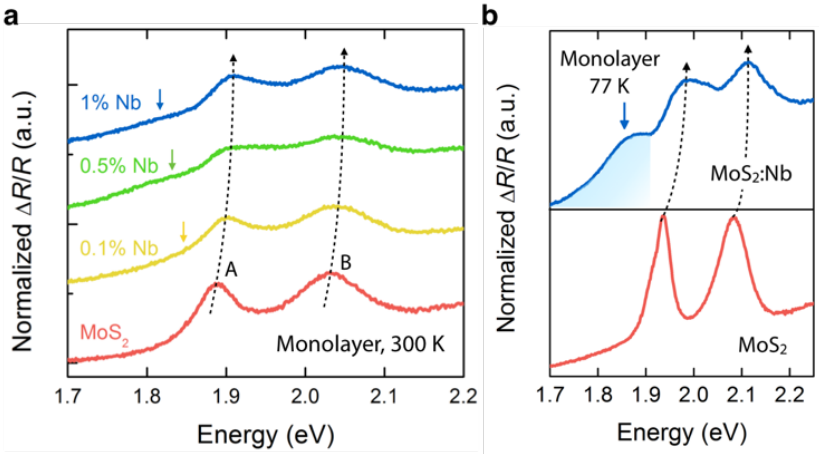

Supplementary Figure 9| Difference reflection spectra ($\Delta R/R$) from monolayer MoS_2 and MoS_2:Nb (**a**) The normalized $\Delta R/R$ for different monolayers measured at 300 K. Corresponding doping levels are indicated, and dotted lines show a blueshift in the A and B exciton levels to guide the eye. (**b**) Representative low-temperature differential reflectivity spectra taken at 77 K from a MoS_2:Nb monolayer with 0.5% of Nb. Blue shade defines the sub-bandgap peak associated with the Nb states.

Supplementary Note 1: Convergent beam electron diffraction

Extensive structural analysis was performed on multiple samples by convergent beam electron diffraction (CBED) in order to determine and distinguish the phase of the synthesized bulk crystals. A total of 51 crystal samples have been investigated. During our investigation, all undoped MoS_2 crystals (9 out of 9) were determined to have 2H stacking ($P6_3/mmc$) regardless of their production origin; chemical vapor transport (top panel in Fig. 1b) and natural resource (SPI supplies, Supplementary Figure 1a). Upon niobium (Nb) doping, the CBED patterns clearly display 3R features with fewer mirror symmetries (R3m). Only a small fraction of Nb-doped MoS_2 flakes (3 out of 42, ~7%) possesses the 2H stacking, and this small fluctuation does not invalidate our conclusion of the stacking restructuring, given the fact that even undoped MoS_2 crystals were reported to consist of ~10% 3R or mixed phase of 2H and 3R[5]. In addition to the representative image (Fig. 1b in a main text) recorded from 0.5% Nb-doped MoS_2 (MoS_2:Nb) crystal, Supplementary Figure 1b and c correspond to 0.1% and 1% doped samples, respectively, confirming the 3R structure of MoS_2:Nb at all degenerate doping levels we employed.

Supplementary Note 2: Low-temperature reflectance spectrum from the surfaces of bulk crystals

Supplementary Figure 3 shows the reflectance spectrum of bulk MoS_2 and Nb-doped MoS_2 (MoS_2:Nb) at 4.5 K. The observed dominant transitions in the spectrum were fit starting with a dielectric function $\varepsilon(E)$ of the form

$$\varepsilon(E) = \varepsilon_{\mathrm{b}} + \frac{C}{E_0^2 - E^2 - i\Gamma E} \quad (1),$$

where ε_b is the background dielectric constant and the rest is the contribution from a particular exciton transition according to the Lorentz oscillator model[6]. Here E is the photon energy while E_0, Γ, and C are the transition energy, homogeneous broadening and amplitude parameter for the transition, respectively. The exciton contribution to $\varepsilon(E)$ was further convolved with a Gaussian to account for the large inhomogeneous broadening[7]. For undoped MoS_2 crystal, the transition energies obtained through fitting using equation (S1) are 1.916, 1.958 and 2.115 eV and 2.143 eV. These can be identified as A_K, A_H, B_K and B_H transitions respectively, of the most common 2H polymorph of MoS_2[6]. The B_K and B_H transitions are unresolved here but can be distinguished in a modulated reflectance measurement[6]. In contrast, most of MoS_2:Nb crystals only show distinct A_K (B_K) of 1.904 (2.056) eV implying its 3R stacking. No separate H-point transition is observed although the A_K still has reasonably narrow width. Additionally, we note that some parts of MoS_2:Nb have a slightly different spectrum as shown in Supplementary Figure 3c. It also displays two main transitions (A_K and B_K), redshifted A_K and no H-point feature like 3R phase. The energy splitting, E_B-E_A, is however larger than that for 3R although it is not as large as that of 2H. Therefore, it can be regarded as the intermediate phase between 2H and 3R of MoS_2, presumably mixed stacking sequences considering their broad features and median nature[5].

Supplementary Note 3: Absorption spectra of undoped and Nb-doped MoS_2 monolayer

Opposite to the redshift in PL spectra, absorption spectra reveals a slight blueshift in the A and B exciton energies in MoS_2:Nb monolayers, as recorded using differential reflectance and shown in Supplementary Figure 5. They also possess a slight dependence on the doping level, and the shift reaches ~20 meV at the highest doping concentration of 1%. In addition to these main excitonic peaks, a long low-energy tail (arrows in Supplementary Figure 5a) was observed for all monolayer MoS_2:Nb samples. Lower temperatures allow clearer detection of the absorption shoulder at ~100 meV below the A exciton resonance, which is in agreement in energy with the impurity-mediated PL peak shown in Figure 4a. In order to understand the slight blueshift of the A and B exciton peaks, we first consider the Burstein-Moss (B-M) shift, as done for other, heavily doped MoS_2 system[8]. The B-M shift is the blueshift in absorption energy across the fundamental bandgap of a degenerately n (p)-type doped semiconductor because the edge of its conduction (valence) band is no longer completely empty (filled) and hence fully accessible for optical transitions. Quantitatively, using $\Delta E_{\text{B-M}} = (\hbar^2/2m_{\text{p}}^*)[3\pi^2(p/N_{\text{K}})]^{2/3}$ where m_{p}^* is the effective hole mass, N_{K} is the valence band multiplicity factor at the K point (6), and p is carrier density previously determined by Hall-effect measurements[9], the B-M shift $\Delta E_{\text{B-M}}$ is estimated to be ~19 meV for 0.5% doping of Nb. Although the estimated value is comparable to the observed blueshift, this effect alone cannot explain the observation: the B-M shift expects a blueshift only in the lowest transition (*i.e.*, the A exciton), while we observed simultaneous blueshift in both the A and B exciton energies in spite of an energy separation of nearly 150 meV between them.

Additionally, the existence of dense hole gas distributed in a monolayer thickness cannot be ignored, since it effectively screens the binding energy of excitons, as recently observed in electrostatically gated[10] and theoretically modelled heavily doped monolayer MX_2[11], both showing blueshifts of exciton resonances. Considering that our nominal 2D hole density (~1.2×10^{13} cm^{-2} for 1% Nb-doped MoS_2) exceeds the maximum carrier density (~8×10^{12} cm^{-2}) previously achieved by field-effect gating, plus additional dielectric screening from charged impurity centres, it is reasonable to expect even stronger effects compared to the reported binding energy reduction in Ref. [1]. However, more investigations are needed along with a quantitative evaluation of bandgap renormalization effects, which tends to counteract the reduction of exciton binding energy.

Supplementary References

1. Suzuki, R. *et al.* Valley-dependent spin polarization in bulk MoS_2 with broken inversion symmetry. *Nat. Nanotechnol.* **9,** 611–617 (2014).
2. Togo, A and Tanaka, I. First principles phonon calculations in materials science. *Scr. Mater.* **108,** 1–5 (2015).
3. Shmeliov, A. *et al.* Unusual stacking variations in liquid-phase exfoliated transition metal dichalcogenides. *ACS Nano* **8,** 3690–3699 (2014).
4. Lin, Y.-C. *et al.* Properties of individual dopant atoms in single-layer MoS_2: atomic structure, migration, and enhanced reactivity. *Adv. Mater.* **26,** 2857–2861 (2014).
5. Lee, J.-U. *et al.* Raman signatures of polytypism in molybdenum disulfide. *ACS Nano* **10,** 1948–1953 (2016).
6. Saigal, N. & Ghosh, S. H-point exciton transitions in bulk MoS_2. *Appl. Phys. Lett.* **106,** 182103 (2015).
7. Saigal, N., Sugunakar, V. & Ghosh, S. Exciton binding energy in bulk MoS_2: a reassessment. *Appl. Phys. Lett.* **108,** 132105 (2016).
8. Sun, Q. C. *et al.* Observation of a Burstein–Moss shift in rhenium-doped MoS_2 nanoparticles. *ACS Nano* **7,** 3506–3511 (2013).
9. Suh, J. *et al.* Doping against the native propensity of MoS_2: degenerate hole doping by cation substitution. *Nano Lett.* **14,** 6976–6982 (2014).
10. Chernikov, A. *et al.* Electrical tuning of exciton binding energies in monolayer WS_2. *Phys. Rev. Lett.* **115,** 126802 (2015).
11. Gao, S., Liang, Y., Spataru, C. D. & Yang, L. Dynamical excitonic effects in doped two-dimensional semiconductors. *Nano Lett.* **16,** 5568–5573 (2016).
